# Characteristics of Cardiac Ketone Body Metabolism Throughout the Life Cycle

**DOI:** 10.1155/cdr/5913327

**Published:** 2025-05-25

**Authors:** Lei Li, Xiaojian Song, Xiurui Ma, Yanwu Xu

**Affiliations:** ^1^Department of Cardiology, Shan Xi Cardiovascular Hospital, Taiyuan, China; ^2^Department of Biochemistry, School of Integrative Medicine, Shanghai University of Traditional Chinese Medicine, Shanghai, China

**Keywords:** heart, ketone body, metabolism, physiology

## Abstract

Ketone bodies can serve as energy substrates for the heart and perform important molecular signal transduction functions. In recent years, the therapeutic potential of ketone bodies has become a research hotspot in the field of cardiovascular diseases. Many previous reviews have focused on ketone bodies from the perspective of cardiovascular diseases, especially heart failure. Nonetheless, the metabolism of cardiac ketone bodies under physiological conditions also warrants attention, as the consumption of a ketogenic diet or direct supplementation of ketone bodies from exogenous sources has become widely popular among healthy individuals for weight loss. Furthermore, recent clinical studies have shown that under physiological conditions, the level of ketone bodies is positively correlated with the incidence of cardiovascular diseases and mortality. On the basis of the differences in cardiac ketone body metabolism under healthy and disease conditions, in this review, we describe in detail the characteristics of cardiac ketone body metabolism and the significance of elevated circulating ketone body levels throughout the life cycle on physiological states.

## 1. Introduction

Cardiac ketone body (KB) metabolism involves the utilization of KBs, primarily *β*-hydroxybutyrate (*β*-OHB) and acetoacetic acid (AcAc), by the myocardium as an energy source. Studies on cardiac KB metabolism began in the 1940s. In the 21st century, researchers discovered that KBs are not only energy substrates but also signalling molecules [1–4]. Since 2016, increased cardiac KB metabolism has been reported in heart failure (HF) patients and HF mouse models, triggering a boom in research on KBs in the cardiovascular field [5–7]. Currently, there are numerous reviews on KBs and cardiovascular diseases that outline the changes and therapeutic potential of KBs in various cardiovascular diseases [8–11]. Although KB metabolism presents potential advantages for cardiovascular health, it is imperative to recognize the potential risks associated with its dysregulation. For example, Oyetoro et al. reported that elevated levels of KBs were observed in patients with advanced HF, and higher KB levels were associated with an increased risk of death [12]. Shemesh et al. demonstrated that in 5% of the population, the blood KB levels exceeded the normal range and that elevated KB levels were positively correlated with the incidence of cardiovascular diseases and mortality [13]. The role of KBs in cardiovascular health has recently gained significant attention, especially in various physiological and pathological contexts. Cardiac KB metabolism plays a crucial role in heart health, not only in individuals with cardiovascular disease but also in the generally healthy population [8, 14]. We must pay greater attention to the physiological characteristics and effects of cardiac KB metabolism. This study explored the characteristics and significance of cardiac KB metabolism in normal physiological states, elucidated the metabolic features of KBs in cardiomyocytes at various stages of growth and development, and examined the factors that influence KB metabolism.

## 2. KB Metabolism

### 2.1. Ketogenesis

Circulating KBs are a group of three compounds, including *β*-OHB, AcAc, and acetone [15, 16]. KBs are synthesized in the liver, largely from circulating free (nonesterified) fatty acids. In hepatocytes, fatty acids are converted to long-chain acyl-CoA and undergo *β*-oxidation in mitochondria, which produces acetyl-CoA (Ac-CoA). In carbohydrate-limited, catabolic conditions with increased gluconeogenesis, insufficient oxaloacetate is available for combination with Ac-CoA, which is instead converted to acetoacetate and then reduced to *β*-OHB in an NAD+/NADH-coupled equilibrium reaction that is catalysed by mitochondrial *β*-OHB dehydrogenase 1 (BDH1) [14]. Finally, KBs are released by the liver into aqueous plasma via solute carrier 16A (SLC 16A) and monocarboxylate transporter Family Members 1, 6, and 7 and circulate to extrahepatic tissue for terminal oxidation [4].

### 2.2. Cardiac KB Metabolism

The human heart can produce approximately 6 kg (almost 12 times its own weight) of ATP every day. In a normal adult heart, fatty acids are the main fuel for mitochondrial ATP production, providing 40%–70% of the ATP supply, followed by glucose (20–30%), lactic acid (5–20%), and KBs (5–15%), among other energy sources [17]. The myocardium is one of the tissues that is capable of metabolizing KBs [9, 18]. Under conditions of increased KBs, a normal heart preferentially uses KBs to provide energy, while both fatty acid oxidation (FAO) and glucose oxidation are reduced [19].

The uptake and utilization of KBs by cardiomyocytes are the results of the coordinated action of multiple enzymes, making the process complex. KBs are transported from the circulation to myocardiocytes and mitochondria via SLC16A1 and SLC16A7 [20]. Within mitochondria, BDH1 catalyses the oxidation of d-*β*OHB to AcAc. AcAc is activated to AcAc-CoA by succinyl-CoA:3-oxoacid-CoA transferase (SCOT) [21]. A reversible AcAc-CoA thiolase reaction yields two molecules of Ac-CoA that enter the TCA cycle [5, 22]. Studies on many organisms, including humans, have shown that the heart has a high oxidative capacity for KBs [23, 24].

## 3. The Characteristics and Significance of Cardiac KB Metabolism in the Foetal Period, After Birth, and During Ageing

### 3.1. Foetal Cardiac KB Metabolism

During the foetal period, the heart uses lactic acid and glucose as its main energy substrates, and long-chain fatty acids and KBs usually cannot be used as energy substrates [18]. Therefore, the foetal heart poorly metabolizes KBs.

During pregnancy, a woman's body undergoes a series of significant changes. Her metabolism increases, her hormone levels shift, her glucose consumption decreases, and she begins to rely on fat reserves for energy [25]. Moreover, in pregnant women, fat is broken down to provide a large quantity of fatty acids in the circulation, and the oxidation of fatty acids by liver cells increases to generate a large amount of Ac-CoA, which is converted into KBs by the liver [26]. Theoretically, pregnant women may be more prone to ketosis than nonpregnant women because of physiological changes during pregnancy. Paterson et al. studied the blood KB levels and urine KB levels of 22 healthy pregnant women and reported that the mean plasma KB level in pregnant women, 0–43 *μ*mol/mL, was considerably greater than that of 0–13 *μ*mol/mL in nonpregnant women [27]. Ketosis is indeed more likely to occur during pregnancy than in nonpregnant states, which is supported by both theoretical findings and experimental results. Because KBs can enter the foetus through the placenta [28], maternal blood KB levels are closely related to foetal plasma KB levels [27, 29]. Owing to the weak ability of foetuses to metabolize KBs, ketoacidosis in pregnant women is a dangerous condition. Metabolic disorders caused by ketosis can lead to foetal hypoxemia and acidosis and even cause foetal loss. Research has shown that if the foetal beta hydroxybutyrate level increases from 0.12 ± 0.08 to 6.80 ± 0.46 mmol/L, it is associated with a significant decrease in foetal PaO2, suggesting that foetal oxygenation is significantly reduced during hyperketonaemia and may contribute to increased perinatal mortality [30]. In one case report, foetal death occurred with a maternal blood KB concentration of 9.2 mM and a foetal blood KB concentration of 4.2 mM, suggesting that foetuses are less tolerant to high blood KB levels [31]. A case report noted that in pregnant women with hyperketonaemia, their foetuses had an increased heart rate, reduced variability, and deceleration; after the resolution of metabolic disorders, the foetal heart rates normalized [32]. Compared with pregnancies in which the KB levels are normal, animal models of pregnancy with high KB levels presented more cardiac malformations. When pregnant mice were fed a ketogenic diet (KD), the embryos presented varying heart and brain volume percentages compared with those of pregnant mice fed a standard diet. These growth changes may be a result of embryonic and organ energy substrate preferences, which are modulated by their concentrations in maternal circulation, as well as their transport across the placenta at various gestational time points [33].

Although the foetal heart is intolerant to KBs and ketosis is more harmful in utero, the specific mechanism is still unclear. Studies have shown that the foetal heart has a low mitochondrial content and low tricarboxylate cycle enzyme and electron transfer chain enzyme activities; therefore, the ability of the foetal heart to metabolize KBs is very weak because it relies on glycolysis as the source of ATP production [34]. The uterus, which contains the foetus, is a hypoxic environment [35]. During chemically simulated hypoxia in vitro, the administration of KBs inhibited myocardial glucose uptake, increased oxidative stress, and aggravated myocardial cell damage [36]. Therefore, in the special environment of the uterus, the foetal heart has a lower tolerance to KBs, leading to a significant increase in damage caused by ketosis.

**Figure 1:**
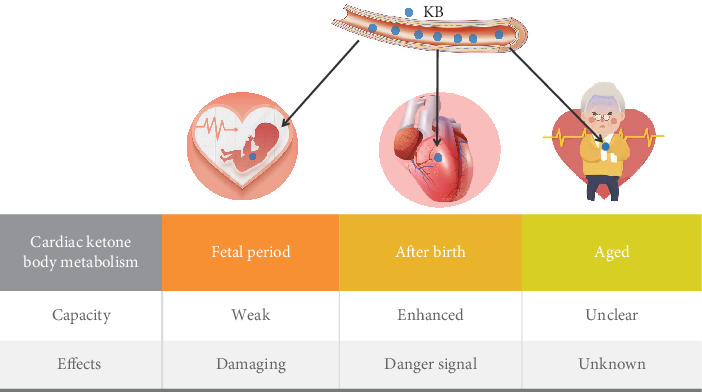
The capacity and effects of cardiac ketone body metabolism in the foetal period, after birth, and during aging. Capacity pertains to the ability of the heart to metabolize ketone bodies; effects describe the impact of elevated serum ketone body levels on cardiac function. KB: ketone body.

In summary, the special metabolic pattern in pregnant women can increase the number of circulating KBs, and the foetal heart has the ability to metabolize KBs. However, the metabolic ability of the foetal heart is weak (Figure 1), and the foetal heart has poor tolerance to hyperketonaemia. KBs have damaging effects on the foetal heart (Figure 1). Given the significant impact that elevated circulating KBs can have on foetal development and the limited understanding of the underlying mechanisms, this area represents a promising field for future research. Studies focusing on the detailed mechanisms of KB-induced damage and potential protective strategies are essential for improving maternal and foetal health outcomes.

### 3.2. Cardiac KB Metabolism After Birth

After birth, owing to changes in cardiac substrates and oxygen availability, the heart switches from relying mainly on glycolysis as a source of ATP to relying mainly on FAO [37]. In the normal adult heart, KBs are an important source of fuel for the production of ATP [14]. The utilization of KBs in the heart depends on the concentration of KBs in the circulation. Cardiac KB oxidation increases with increasing circulating KB supply [18].

Using myocardial oxygen consumption as an indicator of energy substrate utilization, KBs were shown to contribute 5%–10% of the total ATP in the hearts of healthy individuals [38]. Studies by Horton et al. revealed that KBs account for approximately 10%–15% of ATP production in dog hearts [39]. Murashige et al. used metabolomics on blood from the coronary sinus in 110 patients with or without HF and reported that KBs consumed by the heart are positively proportional to the level of KBs in the circulation [23]. In another study, after aortic stenosis, the heart was perfused ex vivo; when the *β*-OHB concentration increased from 0.2 to 0.6 mM, the contribution of KBs to heart energy increased to 18%, and total energy production increased by 23% [40]. Ex vivo perfusion of mouse hearts revealed that increasing *β*-OHB concentrations increased myocardial KB oxidation rates without affecting glucose or FAO rates when the physiological levels of glucose (5 mM) and fatty acids (0.8 mM) were normal. Notably, KBs became the major fuel source for the heart at 2.0 mM *β*-OHB, and the increase in the KB oxidation rate significantly increased the activity of the TCA cycle [41]. However, these two studies also revealed that although increased cardiac KB metabolism increases the total energy production of the heart, myocardial oxygen consumption also increases; therefore, an increase in cardiac KB metabolism is not accompanied by an increase in cardiac work. Studies at the mitochondrial level in vitro have shown that KBs are not the main source of myocardial energy production [42]. Therefore, although KBs can effectively produce ATP, they are not an essential energy source for the heart.

Elevated KBs in the general population may be a pathological mediator, potentially leading to adverse health effects, including heart damage. Shemesh et al. reported that elevated KBs were positively correlated with the incidence and mortality of cardiovascular diseases [13]. Obokat et al. reported that increased *β*-OHB levels were independently associated with cardiovascular events and all-cause death in 405 haemodialysis patients [43]. The results of a population-based study by Flores-Guerrero et al. revealed that high plasma *β*-OHB levels are associated with an increased risk of HF with a reduced ejection fraction (HFrEF), especially in women [44]. The results of these two studies both indicated that elevated KBs were positively associated with increased cardiovascular risk.

In summary, after birth, the capacity for KB oxidation in the heart increases. KBs can effectively produce ATP to provide energy for the heart (Figure 1), but they are not an essential source. Although KBs serve as significant sources of fuel for ATP production, their levels in the heart under physiological conditions are relatively low, and their contribution to the overall oxidative metabolism of the heart remains minimal. In the general population, elevated KB levels could serve as a potential biomarker for cardiovascular risk assessment (Figure 1). The mechanism by which elevated KB levels affect a healthy heart requires further study.

### 3.3. Cardiac KB Metabolism During Ageing

In the ageing heart, the metabolic pattern of myocardial tissues changes, accompanied by changes in the structure and function of the heart [45]. The changes in substrate metabolism in the ageing heart mainly manifest as impairments in FAO and an imbalance in glucose utilization [45]. In previous studies, the ageing myocardium was shown to have significant insulin resistance, thus affecting glucose uptake and fatty acid metabolism [46]. In the cytoplasm in ageing myocardial cells, both the number of mitochondria and the oxidative phosphorylation ability decrease, whereas the generation of oxygen free radicals increases [47]; all these changes in ageing myocardial cells affect cardiac metabolism.

The extent of plasma AcAc elevation in adult rats (50 weeks old) was significantly lower than that in young rats (8 weeks old) following a 36-h fasting period [48]. Moreover, Hernandez et al. reported that aged rats (20 months) need more time than young rats (4 months) do to reach asymptotic levels of plasma *β*-OHB when fed a KD [49]. Research suggests that older adults may have a diminished capacity for KB production, which is likely attributed to reduced fatty acid mobilization and altered enzyme activity within the ketogenesis pathway [50]. A study conducted by Hernandez et al. demonstrated that aged rats exhibit a delayed response to KDs compared with their younger counterparts, which is associated with hyperinsulinaemia [51]. On the basis of the aforementioned studies, whether through starvation or the consumption of a KD, the adaptation process of energy substrates transitioning from glycolysis to keto-oxidation is delayed with increasing age, and several other researchers have agreed with this [52]. However, Hagopian et al. reported that the total number of KBs in the livers of aged caloric-restricted mice was significantly greater than that in the livers of older control mice; however, the underlying mechanism was not investigated [53]. 

Hyyti et al. performed isolated heart perfusion on aged mice (22–24 months) and administered ^13^C-labelled free fatty acids, acetoacetate, lactic acid, and glucose and reported that although the hearts of aged mice still had the ability to metabolize fatty acids and KBs, their metabolic ability was obviously disrupted [54]. However, Rebrin et al. reported that in heart mitochondria, there was no alteration in the total content of SCOT, a key enzyme of KB catabolism, but the specific activity of SCOT increased in 24-month-old rats compared with 4-month-old rats [55]. Thus, it remains unclear whether KB metabolism is decreased or increased in aged hearts. The extent to which cardiac KB metabolism changes and its implications for cardiac function in older adults remain areas of active investigation.

Niezen et al. conducted a follow-up study on 1,758 middle-aged and elderly individuals aged 65 years and older without HF over 20 years and revealed a dose–response relationship, with a 50% increase in all-cause mortality between the lowest and highest quintiles of KB concentrations [56]. This association with the elderly population aligns with other studies indicating that elevated levels of KBs may act as a prognostic marker for adverse health outcomes in the general population. For example, a recent study by Post et al. revealed that circulating KBs were associated with all-cause mortality in a general population, emphasizing the need to investigate the underlying mechanisms of this relationship further [57]. Dysregulated KB metabolism may serve as a biomarker for cardiovascular risk. The role of KBs in cardiovascular health is complex and influenced by factors such as dietary patterns, metabolic disorders, and the concentrations of KBs in the circulation [58, 59]. Future research should focus on elucidating the mechanisms underlying these relationships and optimizing dietary strategies to leverage the cardioprotective effects of KB metabolism.

In summary, the ability of the heart to metabolize KBs in elderly individuals remains unclear (Figure 1). Additionally, the effects of KBs on the hearts of elderly individuals are unknown (Figure 1). The exploration of KB metabolism in the ageing heart is essential not only for understanding the underlying mechanisms of age-related cardiac dysfunction but also for identifying potential therapeutic interventions. Future research should be aimed at clarifying the role of KBs in cardiac metabolism and their implications for treatment strategies in the elderly population.

## 4. Factors Affecting Cardiac KB Metabolism Under Physiological Conditions

### 4.1. Effects of Energy Substrates on Cardiac KB Metabolism

The energy metabolism of the heart is highly flexible. Glucose, fatty acids, KBs, and branched-chain amino acids (BCAAs) compete with each other in the tricarboxylic acid cycle to become the source of Ac-CoA. Therefore, energy substrates affect cardiac KB metabolism.

**Figure 2:**
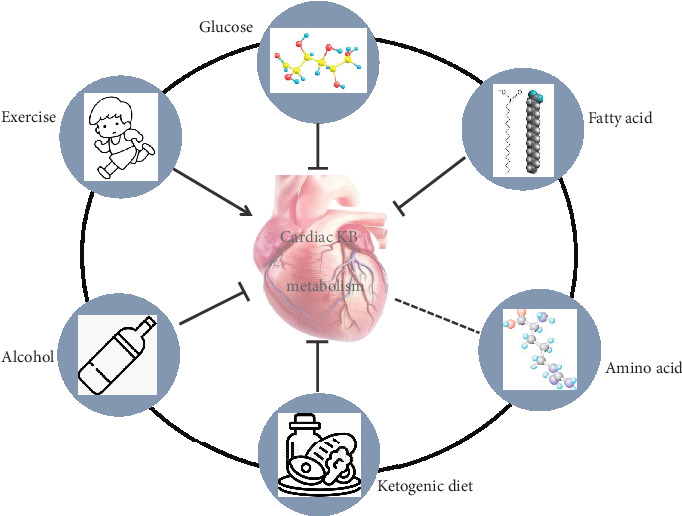
Glucose, fatty acid, and ketogenic diet and alcohol consumption inhibit cardiac KB metabolism; exercise promotes cardiac KB metabolism, and the effects of amino acids on cardiac KB metabolism remain unknown. KB: ketone body.

Studies have shown that myocardial KB metabolism increases when carbohydrate availability is limited [6, 14, 60]. In a study by Janardhan et al., in advanced HF, where glucose oxidation is impaired, the heart adapts by enhancing the oxidation of KBs as an alternative energy source [61]. Some scholars have noted that increased myocardial KB metabolism may be a part of myocardial metabolic remodelling, which changes the utilization of substrates to nonfatty acid sources [7, 62]. This metabolic remodelling is crucial, as it allows the myocardium to maintain energy production under adverse conditions. Under conditions of high glucose availability, the heart tends to prioritize glucose oxidation over FAO, leading to a decrease in KB utilization (Figure 2). In murine models of Type 1 diabetes mellitus (T1DM) and high-fat diet-induced glucose intolerance, elevated blood glucose levels are correlated with reduced cardiac protein levels of BDH and SCOT transferase [63]. Transcriptomic analysis in patients with Type 2 diabetes mellitus and HF confirmed the suppression of BDH1 and OXCT1 gene expression in the heart, which is consistent with findings in mouse models [64]. The suppression of these key cardiac enzymes suggests a shift in cardiac energy metabolism, where the ability of the heart to utilize KBs is impaired. 3-Hydroxy-3-methylglutaryl-CoA synthase 2 (HMGCS2) was the most highly upregulated gene in the hearts of individuals with T1DM, indicating its role in ketogenesis and metabolic pathways associated with T1DM-induced cardiac dysfunction [65]. In the hearts of individuals with T1DM, the transcription and activity of the ketogenesis rate-limiting enzyme HMGCS2 are highly upregulated, the expression of the ketolytic enzymes BDH1 and SCOT is decreased, and the level of KBs is increased [66]. Managing blood glucose levels through interventions such as sodium–glucose cotransporter 2 (SGLT2) inhibitors has been shown to increase KB production and utilization [63]. These findings underscore the importance of glucose levels in regulating KB metabolism. KB metabolism also affects glucose metabolism. By conducting dynamic ^11^C-palmitate, ^15^O-H_2_O, and ^18^F-FDG PET/CT studies in healthy populations, Gormsen et al. reported that when *β*-OHB (0.18 g/kg per hour) was injected, myocardial glucose uptake decreased by half (304 ± 97 (SALINE) vs. 156 ± 62 (KETONE), *p* < 0.01) [67]. These findings suggest that under conditions of elevated KB levels, the heart preferentially utilizes ketones over glucose. However, Xu et al. reported that a KD or *β*-OHB injection increased the levels of the glycolytic metabolites pyruvate and lactic acid in normal rat myocardial tissue [68]. After myocardial cells were treated with *β*-OHB, the expression levels of glucose transporter 1 (GLUT1), hexokinase 2 (HK2), 6-phosphofructo-2-kinase/fructose-2,6-biphosphatase 3 (PFKFB3), and pyruvate kinase M1 (PKM1) increased [69]. Transverse aortic constriction (TAC) in C57BL/6 J mice resulted in a 45% decrease in the ejection fraction; under these conditions, increasing *β*-OHB levels increased KB oxidation and total energy production by 23% while also increasing glucose oxidation [40]. The upregulation of glucose oxidation alongside increased KB utilization suggests a coordinated metabolic response that may help to optimize energy production in the failing heart. These findings collectively underscore the importance of understanding metabolic flexibility in the myocardium, particularly in pathological states where energy substrate availability may be compromised. The ability of the heart to switch between glucose and KBs could have therapeutic implications, especially in the management of HF and metabolic disorders.

Increased fatty acid metabolism in the heart can inhibit KB metabolism (Figure 2). Mice fed a 4-week high-fat diet were shown to have increased fatty acid metabolism in the heart and decreased KB metabolism [70]. In a study by Carley et al., when the oxidation of long-chain fatty acids was impaired in the myocardium, short-chain fatty acids were used as the main energy source instead of KBs [71]. When FAO is limited, the concentrations of C_4_OH-carnitine, C2-carnitine, and succinate in myocardial tissues increase, suggesting that the activation of oxidation pathways of KBs increases [5], which is usually observed in cardiometabolic remodelling in individuals with HF and is associated with increased expression of ketolytic enzymes such as BDH1 [7]. KBs are an effective alternative fuel when myocardial fatty acid metabolism is disrupted. When cardiac KB metabolism increases, fatty acid metabolism is affected. In a study by Danny et al., after acetoacetate was added to isolated rat myocardial cells, the FAO rate significantly decreased [72]. Stanley et al. showed that intravenous injection of *β*-OHB inhibited fatty acid uptake and oxidation in vivo [73]. These findings suggest that the heart may preferentially utilize KBs over fatty acids when available, which could serve as a protective mechanism during periods of reduced energy supply or increased energy demand.

Amino acid oxidation is also a potential source for ATP production by the heart, with BCAA oxidation being the best source of amino acid oxidation in the heart [74]. BCAAs, including leucine, isoleucine, and valine, are essential amino acids that can be obtained from only external food sources. BCAA catabolism plays an important role in heart physiology [75]. One study revealed that the chronic exposure of myocardial cells to high concentrations of *β*-OHB (3 mM or more) can lead to the degradation and inactivation of the endosomal proton pump vacuolar H+-ATPase (v-ATPase), thereby causing systolic dysfunction. Supplementation with a mixture of lysine, leucine, and arginine blocked this effect [76]. Therefore, amino acids may improve adverse events in the heart caused by KBs. Moreover, the accumulation of *β*-OHB, especially at concentrations exceeding 3 mM, could also influence energy substrate utilization by the myocardium. The link between amino acid metabolism and KB metabolism is not yet clear (Figure 2). This highlights the need for a deeper understanding of the interplay between KB metabolism and cardiac performance.

### 4.2. Effects of Fasting and the Consumption of a KD and Alcohol on Cardiac KB Metabolism

Under brief fasting conditions, the total KB production rate and oxidation rate are directly related to increases in plasma KB concentrations [77]. Plasma total KB concentrations range from 100 *μ*M after eating to 1 mM after 24 h of fasting [16, 78], and in the case of prolonged fasting, they can increase to 5–7 mM [79]. Studies have shown that increased circulating KBs can alter cardiac KB metabolism; for example, the long-term consumption of a KD or exogenous *β*-OHB can downregulate the expression of the key enzymes of KB metabolism (BDH1 and SCOT) in myocardial cells [68]. Heart muscle demonstrates limited KB utilization in ketotic nutritional states, as evidenced by the transcriptional suppression of SCOT [80]. When mice were fed a KD for 3 weeks, KB levels significantly increased, and BDH1 and SCOT protein expression significantly decreased [81]. These findings suggest that when the heart is exposed to elevated levels of KBs, their utilization is not necessarily increased; instead, they favour a metabolic state that limits ketolysis [80]. This finding is further supported by NMR profiling, which revealed that the myocardial enrichment of glutamate from labelled KBs is diminished during KD consumption, indicating a reduced oxidative metabolism of KBs [80]. Additionally, Shimizu et al. explored the differential impacts of short-term versus long-term KD consumption on metabolic gene expression in heart and skeletal muscle. Their findings indicate that long-term KD consumption leads to the suppression of genes associated with ketolysis while enhancing those related to lipid utilization. Therefore, KD consumption may reduce the metabolism of KBs in the heart (Figure 2), further illustrating the adaptive metabolic response of the heart [82].

Alcohol also affects cardiac KB metabolism. After 40 days of alcohol gavage, the levels of KBs in the blood and tissues significantly increased, but the activities of KB-utilizing enzymes (BDH and SCOT) in the heart decreased. These findings suggest that alcohol can inhibit cardiac KB metabolism (Figure 2) [83]. Alcohol reduces the ability of the heart to metabolize KBs. Ingested alcohol accounts for 90%–98% of all alcohol eliminated from organisms via three known metabolic pathways: the alcohol dehydrogenase (ADH) and aldehyde dehydrogenase (ALDH) systems, the microsomal ethanol oxidation system (MEOS), and the peroxisome catalase system [84, 85]. Under the action of the first two systems, ethanol is first converted to acetaldehyde and then to acetic acid. Acetic acid and CoA form Ac-CoA, which then enters the tricarboxylic acid cycle. On the one hand, a large amount of NADH is produced in this process, which increases the NADH/NAD^+^ ratio and inhibits the tricarboxylic acid cycle. On the other hand, Ac-CoA accumulation also inhibits KB utilization in the myocardium and promotes KB synthesis in the liver, presumably increasing KB levels [85].

### 4.3. Effect of Exercise on Cardiac KB Metabolism

The type and intensity of exercise play critical roles in modulating KB metabolism in the heart. Research has demonstrated that low- to moderate-intensity exercise can increase the levels of circulating KBs, particularly in the context of a KD or exogenous KB supplementation [86]. For example, studies have shown that during aerobic exercise, the body shifts its energy substrate preference from glucose to fatty acids and KBs, especially when glycogen stores are depleted [9]. High-intensity exercise, on the other hand, may result in a more complex metabolic response, where lactate production can temporarily inhibit KB utilization. However, the recovery phase following intense exercise is characterized by significantly decreased circulating *β*-OHB levels, suggesting that the heart may preferentially utilize these substrates during recovery to replenish energy stores and support myocardial function [87]. KBs are oxidized as a fuel source during exercise. A 14-week treadmill running program increased KB utilization enzyme levels in skeletal muscles, with significant increases in 3-hydroxybutyrate dehydrogenase and 3-ketoacid CoA-transferase, and the levels of ketolytic enzymes in the heart did not increase correspondingly [81, 82]. These results suggest that myocardial KB metabolism remains relatively stable during exercise. However, there were differences in cardiac KB metabolism between the sexes during exercise. In female mice subjected to high-intensity exercise, *β*-OHB levels in myocardial tissues increased to 2.2 mM, whereas in males, there were no significant changes [88]. Although the specific mechanism is still unclear, it is speculated that it may be related to oestrogen. For example, 17b-oestradiol may regulate homeostatic levels of metabolites in cardiomyocytes by affecting mitochondrial dynamics [88]. In a study by Shimizu et al., blood levels of 3-hydroxybutyric acid were significantly increased in the KD group, whereas long-term exercise attenuated this increase, suggesting that exercise may have a regulatory effect on the metabolic effects of a KD [82]. Collectively, these findings suggest a complex interplay among exercise, hormonal factors, and metabolic pathways that warrants further investigation to elucidate the underlying mechanisms involved. Exercise training may enhance the ability of cardiac muscle to utilize KBs (Figure 2), and this adaptation could improve cardiac efficiency and function; however, detailed studies are needed to elucidate these mechanisms.

## 5. Summary and Prospects

Cardiac KB metabolism is a dynamic area of research that reveals the remarkable ability of the heart to adapt to varying metabolic demands throughout different life stages—from foetal development to adulthood and into old age. Cardiac KB metabolism is also affected by a variety of factors, such as energy, fasting, KD consumption, alcohol consumption, and exercise. In recent years, although there has been much evidence of the beneficial effects of KBs on the heart, some clinical studies have noted the negative effects of KBs on the heart. In clinical practice, to effectively use KBs in cardiovascular applications, it is necessary to conduct a detailed study on cardiac KB metabolism during different periods under physiological conditions. At present, many regulatory mechanisms are still unclear. Understanding cardiac KB metabolism is essential for understanding the energy dynamics of the heart and developing potential interventions for metabolic and cardiovascular diseases. Continued research in this area promises to unlock new insights into cardiac function and pave the way for novel therapeutic approaches that harness the benefits of KBs.

## Data Availability

All data referenced in this review are derived from peer-reviewed publications and open-access repositories, with direct citations provided throughout the manuscript. No original datasets were generated in this work.
